# The Impact of Diabetes on the Labour Force Participation and Income Poverty of Workers Aged 45–64 Years in Australia

**DOI:** 10.1371/journal.pone.0089360

**Published:** 2014-02-20

**Authors:** Deborah J. Schofield, Michelle Cunich, Rupendra N. Shrestha, Emily J. Callander, Megan E. Passey, Simon J. Kelly, Robert Tanton, Lennert Veerman

**Affiliations:** 1 NHMRC Clinical Trials Centre, University of Sydney, Australia; 2 School of Public Health, University of Sydney, Australia; 3 University Centre for Rural Health – North Coast, School of Public Health, University of Sydney, Australia; 4 National Centre for Social and Economic Modelling, University of Canberra, Australia; 5 School of Population Health, University of Queensland, Brisbane, Queensland, Australia; Old Dominion University, United States of America

## Abstract

**Objective:**

To quantify the poverty status and level of disadvantage experienced by Australians aged 45–64 years who have left the labour force due to diabetes in 2010.

**Research Design and Methods:**

A purpose-built microsimulation model, Health&WealthMOD2030, was used to estimate the poverty status and level of disadvantage of those aged 45–64 years who prematurely retire from the workforce due to diabetes. A multiple regression model was used to identify significant differences in rates of income poverty and the degree of disadvantage between those out of the labour force due to diabetes and those employed full- or part-time with no diabetes.

**Results:**

63.9% of people aged 45–64 years who were out of the labour force due to diabetes were in poverty in 2010. The odds of being in poverty for those with no diabetes and employed full-time (OR of being in poverty 0.02 95%CI: 0.01–0.04) or part-time (OR of being in poverty 0.10 95%CI: 0.05–0.23) are significantly lower than those for persons not in the labour force due to diabetes. Amongst those with diabetes, those who were able to stay in either full- or part-time employment were as much as 97% less likely to be in poverty than those who had to retire early because of the condition. Sensitivity analysis was used to assess impacts of different poverty line thresholds and key socioeconomic predictors of poverty.

**Conclusions:**

This study has shown that having diabetes and not being in the labour force because of this condition significantly increases the chances of living in poverty. Intervening to prevent or delay the onset of diabetes is likely to improve their living standards.

## Introduction

Diabetes is increasingly common, affecting an estimated 246 million people globally [1]. This figure is expected to reach 380 million by 2025 [2] due to increasing obesity and sedentary lifestyles, and the ageing of the global population [3], [4]. The most recent Burden of Disease report identified diabetes as the second leading cause of burden of disease in men, and the fourth leading cause of burden of disease in women in Australia [5].

Australia, like many countries, has a population that is ageing and thus an increasing proportion of older workers are aged 45–64 years [6], [7]. Diabetes, which has been demonstrated to adversely affect an individual's ability to work [8], is responsible for the early retirement of a large number of individuals within this age group. In Australia, 37.9% of people aged 45–64 years with diabetes are currently not in the labour force [9].

Retiring early due to diabetes has significant national costs, with lost labour force participation being identified as making up a significant proportion of the total costs of diabetes [1], [10], [11], [12], [13], [14]. However, the costs to the individual are significant as well – those who have left the labour force due to diabetes have significantly lower income and savings than those who are in the labour force without this condition [15], [16]. This is likely to markedly reduce the living standards of these individuals due to their poorer financial resources. Examining the poverty status of comparable households is one way of assessing living standards of a defined population, and poverty is used as an indicator of living standards in modern society [17]. Having diabetes may increase the chances of an individual living in poverty due to their lower labour force participation rate and subsequent poorer financial status. However, no studies to date have identified how susceptible individuals with diabetes are to living in poverty, due to the condition's ability to impact on their labour force participation.

This paper will examine the relationship between labour force participation, diabetes and income poverty. It aims to quantify the difference in the likelihood of being in poverty between those who are not in the labour force due to diabetes and those with no chronic health condition in various states of employment. It will also examine the likelihood of being in poverty amongst those with diabetes who are out of the labour force due to their illness, compared to those who are able to continue to work full or part-time. Sensitivity analysis will be used to assess the impacts of different poverty line thresholds and key socioeconomic predictors (education) of poverty. Finally, we will estimate the number of Australians who are not in the labour force due to diabetes who were in poverty in 2010 and compare how being out of the labour force due to diabetes increases the chances of being in poverty compared to those in employment and those out of the labour force for other reasons.

## Methods

### Data

We used Health&WealthMOD2030, an extension of a previous microsimulation model of health, disability and labour force participation we assembled [18], to analyse the impact of diabetes on labour force participation, poverty status and level of disadvantage for workers aged 45–64 years in 2010. Health&WealthMOD2030 was specifically designed to estimate the economic impacts of ill health on the labour force status of Australians aged 45–64 years between 2010 and 2030.

The base population of Health&WealthMOD2030 was unit record data for those aged 45–64 years extracted from two Surveys of Disability, Ageing and Carers (SDACs) conducted by the Australian Bureau of Statistics (ABS) in 2003 and 2009 [19], [20]. These nationally representative household survey data consist of demographic data (such as age, sex, family type and state of residence), socioeconomic data (such as level of education, income and home ownership), labour force data (such as labour force participation, employment restrictions and retirement), and health and disability data (such as chronic conditions, health status, type and extent of disability, support and care required) for each individual in the household.

Respondents in the SDACs reported what their main and other health conditions were, and their responses were classified using ICD10 codes by the ABS [19], [20]. In this study, respondents were considered to be out of the labour force due to diabetes if they stated they were out of the labour force due to their illness and listed diabetes as their main condition.

The combined (2003 and 2009) SDACs data were reweighted to reflect the profile of the 2010 Australian population aged 45–64 years using a reweighting algorithm GREGWT developed by the ABS to reweight their survey data [21]. This reweighting procedure was used to account for the changes in disability and illness, demographics, labour force participation and other features of the population that occurred between the years for which we have data (2003 and 2009) and 2010.

The SDACs included income data presented in ranges. For the purpose of this paper, we derived more detailed information on income from a separate microsimulation model called the Australian Population and Policy Simulation Model (APPSIM) [22]. APPSIM is a cross-sectional, dynamic population microsimulation model that provides a snapshot of the sociodemographic and economic characteristics (such as income and government support payments) of the population annually. It was developed and is maintained by the National Centre for Social and Economic Modelling (NATSEM; www.natsem.canberra.edu.au). Detailed economic information from APPSIM were imputed onto the base population of Health&WealthMOD2030 by identifying persons with similar characteristics on APPSIM and imputing their income and wealth information onto Health&WealthMOD2030 using a process commonly used in microsimulation modelling called synthetic matching [23]. Ten variables that were common to both datasets and strongly related to income were chosen as the matching variables: labour force status (4 groups), income unit type (4 groups), income quintile (5 groups), receiving/not receiving age pension (2 groups), receiving/not receiving disability support pension (2 groups), sex (2 groups), age group (4 groups), hours worked per week (5 groups), highest educational qualification (2 groups) and home ownership (2 groups).

### Defining Reason for Leaving the Labour Force

The SDACs ask respondents about their current labour force status, and if they respond that they are not in the labour force, what the reason for this was, in particular whether they were out of the labour force due to their ill health. Survey respondents' health conditions were classified by the ABS as a part of the survey using ICD10 codes. People who were identified as being out of the labour force due to ill health and who nominated diabetes (ICD10 Codes: E10–14, E74.8, E83.3) as their main health condition were considered to be out of the labour force due to diabetes in this study [19]. Whilst the SDAC data does not distinguish the type of diabetes of respondents, we note that these people are most likely to have Type 2 diabetes based on the age group of study participants [24].

### Poverty lines

A ‘poverty line’ is defined as the level of income below which an economic unit (a person, family or household) is in poverty, that is the unit's level of income is insufficient for purchasing all the goods and services required to maintain a basic standard of living. Poverty lines are usually adjusted to take into account the composition of the economic unit (family or household) using equivalence scales, and expressed in terms of a percentage of the equivalised average or median income.

However, considering only an individual's personal income is not seen as a complete reflection of an individual's economic circumstance. Within a family, it is assumed that members pool their economic resources to the benefit of all members. Thus, poverty status is typically assessed using the aggregated income of all members in the family as this will provide a better estimate [25]. The ‘income unit’ grouping recorded by the ABS on the SDACs were utilised in this study to identify the members of the family that do group their income. The income unit is defined by the ABS as “a group of two or more related persons in the same household assumed to pool their income and savings and share the benefits deriving from them equitably; or one person assumed to have sole command over his or her income, consumption and savings” [19: 6]. The terms ‘income unit’ and ‘family’ are interchangeable in the remainder of this paper.

To identify individuals in the 45–64 year old Australian population that were in poverty in 2010, a poverty line based on 50 per cent of median family income was used. The median family income was estimated using ‘income unit’ or family income in conjunction with OECD-modified equivalence scales [26], [27]. The median equivalised annual family income for all income units was AU$33 429in December 2010. The 50 per cent of median family income (AU $16 714.50) poverty line expresses the economic situation of those in poverty relative to those in the middle of the income distribution. Those who are in poverty will have at most half the income of those in the middle of the income distribution of the population. The 50 per cent of the median family income has been widely used as a poverty line both in Australia and internationally [28], [29], [30].

Differences in number of family members and the composition of families are taken into account by using equivalence scales [31]. The OECD modified equivalence scale [32] is utilised, whereby a value of 1.0 is given to the first adult member (a person aged 15 years and over), a value of 0.5 to each subsequent adult family member, and a value of 0.3 to each child (a person aged under 15 years). This means that a family made up of a single adult has a value of 1, whereas a family of two adults and two children have an equivalence score of 2.1 (1.0+.5+0.3+0.3). The family's income is divided by their equivalence score, thereby equivalising the income and allowing comparisons between families of different sizes.

### Statistical analysis

Survey respondents aged 45–64 years were grouped into one of six groups based on their labour force status: (a) employed full-time with no diabetes as a chronic health condition, (b) employed full-time with diabetes as main chronic condition, (c) employed part-time with no diabetes as a chronic condition, (d) employed part-time with diabetes as main chronic condition, (e) not in the labour force due to diabetes, and (f) not in the labour force for reasons other than ill health. The proportion of those aged 45–64 years who were in poverty in each group was estimated.

Logistic regression models were used to compare the odds of being in poverty for those who were aged 45–64 years and out of the labour force due to diabetes and those with no diabetes (but who may have had other chronic conditions) who were employed full-time, employed part-time or not in the labour force for reasons not related to their health.

A logistic regression model was constructed to examine the difference in the odds ratio (OR) of being in poverty for those employed full-time with diabetes as their main condition and employed part-time with diabetes as their main condition, compared to those out of the labour force due to diabetes. The model was adjusted for age, sex and education. The difference in odds of being in poverty for those employed full-time with no diabetes, those employed part-time with no diabetes, and those out of the labour force for reasons other than ill health, compared to those out of the labour force due to diabetes was also assessed. The regression models were also adjusted for age, sex and education.

A distributional analysis was conducted for those not in the labour force due to diabetes, not in the labour force due to reasons other than ill health, employed part-time with no diabetes, and those employed full-time with no diabetes. This distributional analysis was undertaken on the equivalised (income unit) income by identifying the proportion of individuals in each group in each income quartile, and representing this information graphically using box and whisker plots.

### Sensitivity Analysis

We undertook two sets of sensitivity analyses. One involved estimating the OR of being in poverty for workers with and without diabetes where the poverty line was based on (a) 50 per cent of median equivalised family income which is commonly used in Australian and North American studies (stated above), and (b) a poverty line based on 60 per cent of median equivalised family income which is the “at-risk-poverty threshold” commonly used in European studies. [33] For the latter, the poverty line (annual income) was estimated to be AU $20 057.40 for Australians in December 2010. The second sensitivity analysis involved estimating the OR of being in poverty (defined as being below the 50 per cent of median equivalised family income threshold) adjusting for (a) age, sex and education vs (b) adjusting for age and sex only.

## Results

In the combined 2003 and 2009 SDACs, there were 25 104 records representing individuals aged 45–64 years. Of these, 12 161 were employed full-time with no diabetes; 4 960 were employed part-time with no diabetes; 5 275 were not in the labour force for reasons other than ill health; 521 were employed full-time with diabetes as their main health condition; 225 were employed part-time with diabetes as their main health condition; and 46 were out of the labour force due to diabetes. There were 4 933 individuals aged 45–64 years who were identified as living below the poverty line (50% of median equivalised income); once weighted, there were 795 904 individuals aged 45–64 years who were found to be in poverty in 2010 (or 20% of the population).

Table 1 shows the number of individuals in poverty by labour force status. Two poverty lines were used in the analysis: (a) a poverty line based on 50 per cent of median equivalised family income, and (b) a poverty line based on 60 per cent of median equivalised family income. With regard to the former measure, we found that those who are out of the labour force due to diabetes have the largest proportion of individuals in poverty, with almost two thirds –63.9% of individuals being in poverty. Those employed full-time with no diabetes (although they may have other chronic health conditions) have the lowest proportion of individuals in poverty, 3.1%. Amongst those who are out of the labour force, those who have diabetes have a larger proportion of individuals in poverty (63.9%) compared to those who are not in the labour force for other reasons other than ill health (42.6%) – a difference of 19 percentage points. By raising the at-risk of poverty threshold to 60% of median equivalised household income, we found that an even higher proportion of people out of the labour force due to diabetes were in income poverty –77%.

**Table 1:** Proportion of individuals in income poverty with varying labour force and health status, amongst workers aged 45 to 64 years in Australia.

Labour force Status	Survey Records	Weighted Population	Weighted Number in Poverty^b^	Weighted Number Not in Poverty^b^	% in poverty^b^	Weighted Number in Poverty^c^	Weighted Number Not in Poverty^c^	% in poverty^c^
NILF, due to diabetes^a^	46	11 334	7 239	4 095	63.9	8 773	2 560	77.4
NILF for reasons other than ill health	5 275	1 270 048	541 157	728 891	42.6	650 105	619 944	51.2
Employed Part-time no diabetes	4 960	1 031 919	167 718	864 201	16.3	242 885	789 033	23.5
Employed full-time no diabetes	12 161	2 540 439	79 790	2 460 649	3.1	135 449	2 404 989	5.33

a NILF  =  Not in labour force.

b Poverty is defined as being below 50 per cent median equivalised family income i.e. AU $16 714.50.

c Poverty is defined as being below 60 per cent median equivalised family income i.e. AU $ $20 057.40.

Once adjusted for age, sex, and education the likelihood of being in poverty (being below 50 per cent of median equivalised family income) is significantly less for those employed full-time with no diabetes or employed part-time with no diabetes, compared to those people not in the labour force due to diabetes (See Table 2). Those employed full-time with no diabetes are 98% less likely to be in poverty than those not in the labour force due to diabetes (OR 0.02, 95% CI: 0.01–0.04). Similarly, those employed part-time with no diabetes are 90% less likely to be in poverty than those out of the labour force due to diabetes (OR 0.10, 95% CI: 0.05–0.23). Those out of the labour force for reasons other than ill health are 41% less likely to be in poverty than those also out of the labour force but because of their diabetes (OR 0.41, 95% CI: 0.18–0.93). Sensitivity analysis was conducted where we re-estimated the OR of being in poverty but only adjusted for age and sex. The results are similar to those discussed above.

**Table 2:** Odds ratio of being in income poverty^a^ for those with and without diabetes by labour force status, adjusted for age, sex and education – amongst workers aged 45–64 years in Australia.

	OR	95% CI	p-value
Not in the labour force due to diabetes	REFERENCE		
Not in the labour force for reasons other than ill health	0.41	0.18–0.93	0.0335
Employed part-time with no diabetes	0.109	0.05–0.23	<.0001
Employed full-time with no diabetes	0.02	0.01–0.04	<.0001

a Poverty is defined as being below 50 per cent median equivalised family income i.e. AU $16 714.50.

Using a subsample of people with diabetes, we found that those employed part-time or full-time are significantly less likely to be in poverty than those out of the labour force, after controlling for age, sex and education (Table 3). Those with diabetes who are employed full-time are 97% less likely (OR 0.03, 95% CI: 0.01–0.08) to be in poverty, and those employed part-time with diabetes are 83% less likely (OR 0.17, 95% CI: 0.06–0.44) to be in poverty than those not in the labour force due to diabetes.

**Table 3:** Odds ratio of being in income poverty amongst those with diabetes but different labour force participation status – amongst workers aged 45 to 64 years in Australia.

	OR	95% CI	p-value
Not in the labour force due to diabetes	REFERENCE		
Employed part-time with diabetes	0.17	0.06–0.44	0.0003
Employed full-time with diabetes	0.03	0.01–0.08	<.0001

**Figure 1:**
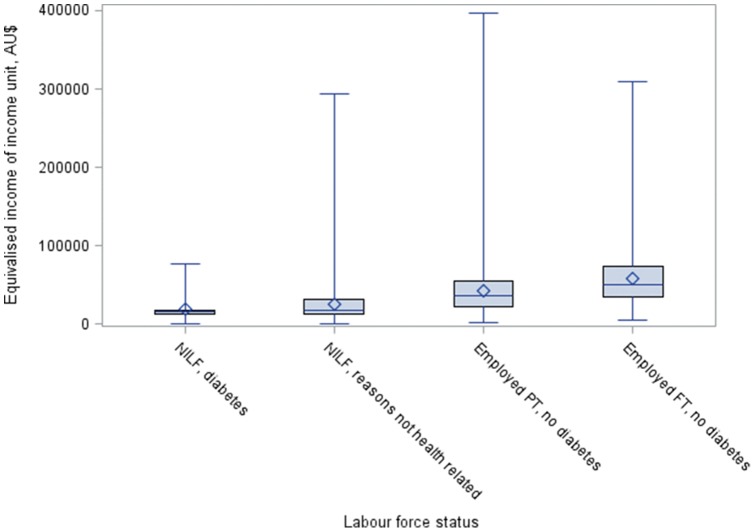
Analysis of the distribution of annual equivalised income for unit incomes by employment status amongst workers aged 45–64 years in Australia.

Figure 1 shows that the annual equivalised income of income units for those not in the labour force due to diabetes is grouped around the lower end of the income distribution –77.4% of those not in the labour force due to diabetes are in the lowest equivalised annual income quartile (as shown in Table 4). The majority of people employed full-time with no diabetes are distributed at the higher end of the income distribution –77.9% are in quartile 1 [highest] and quartile 2 [second highest]. Those employed part-time are more evenly spread across the quartiles.

**Table 4:** Proportion of individuals in each income quartile (equivalised annual income unit income) by employment status amongst workers aged 45–64 years in Australia.

Income quartiles^a^	Employment status			
	NILF due to diabetes	NILF due to reasons other than ill health	Employed part-time, no diabetes	Employed full-time, no diabetes
Q1	77.4%	50.8%	23.3%	5.3%
Q2	13.6%	27.1%	35.3%	25.0%
Q3	3.0%	13.2%	22.4%	35.8%
Q4	6.1%	8.9%	19.1%	33.9%

a Q1 is the lowest income quartile and Q4 is the highest income quartile.

Those not in the labour force due to diabetes had the lowest level of income dispersion, being clustered in the lowest incomes (Figure 1). Only 9.1% of individuals who had retired early due to diabetes were in the top half of the income distribution. Those not in the labour force due to diabetes had a median annual income (income unit) of $15 627 (and annual incomes between the 25^th^ and 75^th^ percentiles of $9 394–$17 782). By contrast, those employed full-time with no diabetes had a higher median income of $49 275 and incomes dispersed over a wider range (incomes between the 25^th^ and 75^th^ percentiles of $35 199–$69 277), followed by those employed part-time with no diabetes with a median income of $33 078 (incomes between the 25^th^ and 75^th^ percentile of $20 643–$52 335).

## Discussion

The likelihood of being in poverty varies with labour force status for those with diabetes. Persons out of the labour force due to diabetes are more likely to be in poverty than any other comparator groups analysed in this study. Amongst those with diabetes, those who were able to stay in either full- or part-time employment are significantly less likely to be in poverty than those who have had to leave the workforce because of the condition. This highlights the importance of labour force participation for those with diabetes to maintaining living standards.

Other studies have been undertaken that have examined the costs of lost productivity and work absence of diabetes [1], [12], [34], [35], [36], [37], [38]. Similarly, Schofield *et al* [39] reported the costs of early retirement due to diabetes, and the subsequent loss of income, and savings [16]. This paper added to this literature by identifying how susceptible individuals with diabetes are to living in poverty, due to the condition's ability to impact on their labour force participation.

It is possible that those who have left the labour force because of their diabetes had little choice in the timing of their retirement. As such, those who retired early because of their diabetes may have had inadequate time to plan their retirement, especially in terms of ensuring adequate financial resources in retirement.

Insufficient income is a known impediment to accessing treatment for diabetes [40], [41]: those in income poverty with diabetes may not be able to access appropriate care and manage their condition in a similar way to those with a higher income. As such, their lower income may exacerbate their health condition and prevent them from remaining in or re-entering the labour force.

There is also evidence that persons who experience diabetes complications (such as blindness, kidney failure, and amputations) are more likely to have a substantial number of additional days absent from work [42], which may impact adversely on their ability to maintain paid employment/find another job if they lose one. Australia has the second highest rate of amputations related to diabetes in the developed world, after the United States [43]. Thus the challenges Australia faces in terms of diabetes management may make it even more likely for persons with diabetes to end up in poverty.

Whilst it has not been possible to identify the length of time people have had diabetes, the nature of their diabetes control (treatment and management) or complications related to diabetes in the 2003 and 2009 SDACs, we note that people with diabetes are more likely to fall into poverty due to insufficient income, savings, lack of home ownership, and other measures of socioeconomic disadvantage than people with other diseases/no disease.

The income distribution analysis has shown that those out of the labour force due to diabetes were mainly clustered around the lower end of the equivalised annual income (all income units) distribution. The income of those out of the labour force due to diabetes was more narrowly dispersed around the lower income end, indicating that the majority of individuals in this group were poor. For those out of the labour force due to diabetes, 77% were in the bottom income quartile. This shows that the vast majority of individuals who have retired early due to diabetes are consistently poor (i.e. are positioned at the lowest end of the income distribution). By comparison, those out of the labour force due to reasons other than ill health had a somewhat wider distribution of income, with 51% being in the bottom income quartile.

This study has shown the detrimental impact that workforce exit due to diabetes has to an individual by significantly increasing their chances of being in poverty. Interventions that prevent or delay the onset of diabetes are likely to improve individual financial situations and hence keep individuals out of poverty. Several studies have demonstrated that interventions preventing (or delaying) the development of type 2 diabetes in high risk individuals are effective [44], [45], [46], [47], [48], [49], [50]. One recent study has shown that increased labour force participation rates can result from lifestyle and metformin interventions [51].
